# Occupational therapy for persons with cognitive impairments

**DOI:** 10.3205/000316

**Published:** 2023-04-04

**Authors:** Petra Schnell-Inderst, Annette Conrads-Frank, Igor Stojkov, Cornelia Krenn, Lisa-Maria Kofler, Uwe Siebert

**Affiliations:** 1 Institute of Public Health, Medical Decision Making and Health Technology Assessment, Department of Public Health, Health Services Research and Health Technology Assessment, UMIT TIROL – University for Health Sciences and Technology, Hall i.T., Austria; 2 Institute of General Practice and Evidence-Based Health Services Research, Medical University of Graz, Austria; 3 Institute for Technology Assessment and Department of Radiology, Massachusetts General Hospital, Harvard Medical School, Boston, MA, USA; 4 Center for Health Decision Science, Departments of Health Policy and Management and Epidemiology, Harvard T.H. Chan School of Public Health, Boston, MA, USA

**Keywords:** technology assessment, biomedical, occupational therapy, cognitive dysfunction, brain damage, chronic

## Abstract

**Background:**

Damage to the central nervous system can occur in adulthood, for example, due to stroke, trauma, tumours, or chronic diseases. After damage to the central nervous system, cognitive impairments occur in addition to physical limitations. Occupational therapy is most often prescribed for neurological diagnoses, including stroke and traumatic brain injury.

**Methods:**

The health technology assessment (HTA) report this HTA article is based on investigates the clinical effectiveness, cost-effectiveness, and patient-related, social and ethical aspects of occupational therapy for patients with cognitive impairments compared to no occupational therapy. In addition, the effects of different occupational therapy interventions with and without cognitive components were compared in an explorative overview. Patients with moderate or severe dementia are excluded from the assessment. Systematic overviews, that is, systematic reviews of systematic reviews, were conducted.

**Results:**

For the evaluation of clinical effectiveness, a total of nine systematic reviews were included. No systematic review was identified for the assessment of costs or cost-effectiveness. Five systematic reviews were included for the assessment of patient and social aspects. For the assessment of clinical effectiveness compared with no occupational therapy, five systematic reviews comprising 20 randomised controlled trials with a total of 1,316 subjects reported small positive effects for the outcomes “global cognitive function” and “activities of daily living” as well as a non-quantified positive effect on the outcomes “health-related quality of life” and “behavioural control”. No effect was found for individual components of cognition and measures of perception. The quality of the evidence for all outcomes is low due to a high risk of bias. In the supplementary presentations, no positive effects could be demonstrated on the basis of the available evidence. The quality of this evidence was not assessed. For the assessment of patient and social aspects, five systematic reviews on patients with a stroke or a traumatic brain injury – without specification regarding cognitive deficits or studies with their relatives – were included. It was reported that patients and family caregivers go through different phases of rehabilitation in which the discharge home is a decisive turning point. The discharge home represents a crucial breaking point. Regaining an active, self-determining role is a process that requires therapists to find the right level of support for patients and relatives. For the assessment of ethical aspects, nine documents were included. We identified ethical problem-solving models for occupational therapy and 16 ethical aspects in occupational therapy for cognitive deficits. The central theme of the analysis is the limited autonomy due to the consequences of the disease as well as the resulting tensions with those treating the patient.

**Conclusions:**

Based on this systematic overview, it can neither be proven nor excluded with certainty that occupational therapy for cognitive impairment is an effective therapy for adult patients with central nervous system injuries compared to no occupational therapy. There is a lack of randomised trials with sufficient sample size, well-defined interventions, and comparable concomitant therapies in the control groups, but there is also a lack of well-designed observational studies in routine care and health economic studies. The identified systematic reviews on patient and social aspects provide information on the needs of patients after stroke or traumatic brain injury and their relatives, but there is a lack of studies on this aspect in German-speaking countries. For the ethical assessment, in addition to the identified theoretical models for solving ethical conflicts in occupational therapy, more empirical studies on ethical aspects with patients with cognitive deficits and their relatives as well as occupational therapists are needed.

## Zusammenfassung

**Hintergrund:**

Schädigungen des zentralen Nervensystems können im Erwachsenenalter zum Beispiel durch Schlaganfall, Trauma, Tumore oder chronische Krankheiten entstehen. Nach Schädigung des zentralen Nervensystems treten neben physischen Einschränkungen auch kognitive Einschränkungen auf. Am häufigsten wird eine Ergotherapie bei neurologischen Diagnosen verordnet, darunter auch Schlaganfall und Schädel-Hirn-Trauma.

**Methoden:**

Der diesem Artikel zugrundeliegende Health Technology Assessment (HTA)-Bericht untersucht die medizinische Wirksamkeit, Kosten und Kosteneffektivität, Patienten- und soziale Aspekte sowie ethische Aspekte der Beschäftigungstherapie für Patienten mit kognitiven Beeinträchtigungen aufgrund von Schädigungen des zentralen Nervensystems im Erwachsenenalter im Vergleich zu keiner Ergotherapie. Ergänzend wurden Formen der Ergotherapie mit und ohne kognitive Komponenten verglichen. Personen mit moderater oder schwerer Demenz sind von der Bewertung ausgeschlossen. Es wurden systematische Overviews erstellt, das sind systematische Übersichtsarbeiten von systematischen Übersichtsarbeiten.

**Ergebnisse:**

Zur Bewertung der medizinischen Wirksamkeit wurden neun systematische Übersichtsarbeiten eingeschlossen. Zu Kosten und Kosteneffektivität konnte keine systematische Übersichtsarbeit identifiziert werden. Für den Vergleich der medizinischen Wirksamkeit gegenüber keiner Ergotherapie wurden in fünf systematischen Übersichtsarbeiten zu 20 randomisierten Studien mit 1.316 Personen kleine positive Effekte für die Zielgrößen „generelle kognitive Funktion“ und „Aktivitäten des täglichen Lebens“ und ein nicht-quantifizierter positiver Effekt auf gesundheitsbezogene Lebensqualität und Verhaltenskontrolle berichtet. Für einzelne Komponenten der Kognition und Zielgrößen der Wahrnehmung ließ sich kein Effekt belegen. Die Vertrauenswürdigkeit der Evidenz für alle Zielgrößen ist auf Grund eines hohen Verzerrungsrisikos gering. In der ergänzenden Darstellung ließen sich anhand der verfügbaren Evidenz keine positiven Effekte nachweisen. Die Vertrauenswürdigkeit der Evidenz wurde nicht bewertet. Zu Patienten- und sozialen Aspekten wurden fünf systematische Übersichtsarbeiten zu Patienten mit Schlaganfall oder Schädel-Hirn-Trauma ohne Angaben zu kognitiven Defiziten oder von deren Angehörigen eingeschlossen. Es wurde berichtet, dass Patienten und betreuende Angehörige verschiedene Rehabilitationsphasen durchlaufen, in denen die Entlassung nach Hause einen entscheidenden Einschnitt darstellt. Die Rückgewinnung einer aktiven, selbstbestimmenden Rolle ist ein Prozess, der den Therapeuten abverlangt, das richtige Maß an Unterstützung von Patienten und Angehörigen zu finden. Zur Bewertung ethischer Aspekte wurden neun Dokumente eingeschlossen. Es wurden ethische Problemlösungsmodelle für die Ergotherapie und 16 ethische Aspekte bei Ergotherapie bei kognitiven Defiziten identifiziert. Zentrales Thema der Analyse sind die eingeschränkte Autonomie durch die Krankheitsfolgen sowie daraus resultierende Spannungen mit den Behandelnden.

**Schlussfolgerungen:**

Auf Grundlage dieses systematischen Overviews kann weder sicher belegt noch ausgeschlossen werden, dass die Ergotherapie bei kognitiven Einschränkungen für erwachsene Patienten mit Verletzungen des zentralen Nervensystems eine wirksame Therapie im Vergleich zu keiner Ergotherapie darstellt. Es fehlen randomisierte Studien mit ausreichenden Fallzahlen, genau definierten Interventionen und vergleichbaren Begleittherapien in den Kontrollgruppen, aber auch gut geplante Beobachtungsstudien in der Versorgungsroutine sowie gesundheitsökonomische Studien. Die identifizierten Übersichtsarbeiten zu Patienten- und sozialen Aspekten geben Aufschluss über die Bedürfnisse von Patienten nach Schlaganfall oder Schädel-Hirn-Trauma und deren Angehörige, jedoch fehlen hierzu Studien im deutschsprachigen Raum. Für die ethische Bewertung werden neben den identifizierten theoretischen Modellen zur Problemlösung ethischer Konflikte in der Ergotherapie mehr empirische Studien zu ethischen Aspekten mit Patienten mit kognitiven Defiziten und deren Angehörigen sowie Ergotherapeuten benötigt.

## Background

This health technology assessment (HTA) article examines occupational therapy for persons with cognitive limitations caused by an injury to the central nervous system in adulthood. Injuries in adulthood can be caused, for example, by a stroke, trauma, tumors or chronic diseases like Parkinson’s disease or multiple sclerosis. These injuries can cause a wide spectrum of symptoms, which may be physical impairments but also cognitive limitations.

Occupational therapy is most commonly prescribed for neurological diagnoses, including stroke and traumatic brain injury [1]. In 2018 the number of stroke diagnoses (ICD-10-codes I61, I63) in German hospitals was 351 cases per 100,000 inhabitants, and the number of cases of traumatic brain injury (ICD-10-code S06) was 343 cases per 100,000 in the same year [2], [3].

According to the definition by the German Society for Psychiatry, Psychotherapy and Neurology (Deutsche Gesellschaft für Psychiatrie, Psychotherapie und Nervenheilkunde) and the German Society for Neurology (Deutsche Gesellschaft für Neurologie), occupational therapy is an intervention for the improvement and support of everyday functions and of the capacity to act with the goal of improvement in participation and in the health-related quality of life within the individual’s daily activities and life context [4]. Occupational therapy employs targeted, individually meaningful activities as an intervention to reduce limitations and improve the patient’s capacity to act independently. In addition, occupational therapy includes the training of specific functions, compensatory measures and consulting for the adaptation of the living environment.

Many interventions in occupational therapy are multimodal and include various therapies targeting the individual needs of each patient. For impairments of the central nervous system, the primarily prescribed interventions are sensomotoric-perceptive treatment, motoric-functional treatment and the neuropsychological-oriented treatment/training of cognitive performance [5].

This HTA article examines the effectiveness, cost-effectiveness, and patient-related, social and ethical aspects of occupational therapy in patients with cognitive limitations. The study excludes patients with moderate to severe dementia. For this population, a DIMDI HTA report has already been published [6].

## Research questions

1. In order to evaluate the clinical efficacy, the following research questions were investigated: how effective are occupational therapy interventions for patients with a cognitive impairment in terms of improving or maintaining cognitive abilities and improving independence, self-determination, and health-related quality of life in inpatient care in social institutions or in outpatient care? Occupational therapy for moderate to severe dementia was not the focus of this article and is, therefore, excluded for all questions.
2. The following research questions were investigated to evaluate economic aspects: does the use of occupational therapy change the resources of inpatient or outpatient nursing and care required for patients with cognitive impairment? Are occupational therapy interventions in the context of inpatient or outpatient care of patients with cognitive impairments cost-effective?
3. The evaluation of patient and social aspects examined the following research questions: which specific patient and social aspects (e.g. experience with the disease, therapy expectations, therapy experience, access to and use of services, cooperation between the doctors, therapists, relatives, and patients) have to be taken into account when using occupational therapy in the context of the care of patients with cognitive impairments?
4. The ethical evaluation examined the following research questions: which ethical aspects on an individual, societal and professional level have to be considered when applying occupational therapy to patients with cognitive impairments?

## Evaluation of clinical efficacy

### Methods

To assess clinical efficacy, a systematic review was conducted based on published systematic reviews from the last ten years.

#### Main inclusion and exclusion criteria for systematic reviews and primary studies

The target population studied includes patients with cognitive impairments caused by diseases of the central nervous system after the age of 18 (remedy guideline 2018 “Heilmittel der Ergotherapie”, Indikation Erkrankungen des Nervensystems 2: “ZNS-Schädigungen nach Vollendung des 18. Lebensjahres” [7]). The intervention studied is occupational therapy compared to no occupational therapy, which may include no other treatment, placebo treatment or other forms of therapy without occupational therapy. In a supplementary presentation, we also examined comparisons of different forms of occupational therapy. The outcome measures sought were the degree of cognitive abilities (attention, memory, thinking, use of language), independence, self-determination, health-related quality of life, and participation in activities of daily living. Systematic reviews, meta-analyses, and HTA reports that summarise RCTs (randomised controlled trial) in a systematic way are included in the systematic overview on clinical efficacy.

#### Literature search, selection, assessment of study quality, data extraction, and evidence synthesis

The bibliographic databases MEDLINE, EMBASE, Cochrane Library, and INAHTA database were searched from January 1, 2010, to May 5, 2020, and October 8, 2020, respectively. The search strategy was composed of three units of free text words and controlled vocabulary from the respective database, joined together with an AND operator. The first unit concerned diseases of the central nervous system, the second unit concerned the intervention searched for, and the third unit restricted the search to systematic reviews, meta-analyses and HTA reports.

The references identified in the search were checked in two stages (titles and abstracts, full texts) by two reviewers for compliance with the inclusion criteria. Conflicting assessments by the two reviewers were resolved through discussion. Reasons for exclusion were documented and the selection process was presented in the form of a PRISMA diagram [8] in the comprehensive HTA report on which this HTA article is based [9]. The identified overviews were searched according to the inclusion criteria of the corresponding systematic reviews.

For reviews that included other types of studies in addition to RCTs, only the description of the RCTs that met the inclusion criteria for population and intervention were relevant for this overview. One reviewer performed the data extraction and a second reviewer checked their accuracy. The characteristics and results of the primary studies were taken from the included reviews.

The assessment of the methodological quality of the included systematic reviews was performed using the AMSTAR 2 tools [10]. For the primary comparison of occupational therapy versus no occupational therapy, the assessment of risk of bias for individual RCTs was also taken from the included reviews.

The evidence synthesis of results concerning clinical efficacy was provided in form of a summary text; in the comprehensive HTA report evidence tables are shown in addition [9]. The final assessment of clinical efficacy was carried out for each pre-specified outcome on the basis of the summarised results of RCTs taken from the systematic reviews.

### Results

#### Clinical efficacy

This overview summarises the evidence of the clinical efficacy of occupational therapy for cognitive impairment from published systematic reviews across indications. A summary of clinical efficacy across varying causes of illness seems justified because occupational therapy is often delivered in mixed groups with patients who had a stroke and patients with TBI. This suggests that similar limitations are present and similar types of therapy are used. The available evidence is based on clinical trials with patients after a stroke, with an acquired brain injury, with Parkinson’s disease or with multiple sclerosis.

Primarily, this overview compares the clinical efficacy of occupational therapy as opposed to no occupational therapy. For this comparison, five systematic reviews [11], [12], [13], [14], [15] with 20 RCTs, comprising a total of 1,316 patients, were included. 

A small positive effect of occupational therapy for patients with cognitive impairment compared to no occupational therapy is reported from a meta-analysis with nine RCTs with 708 subjects [13]. For a combination of different outcome measures (cognitive outcome measures, activities of daily living, value attitudes) the effect is measured as standardized mean difference (SMD) Cohen’s d. Its magnitude is 0.19 (95% CI: 0.10; 0.23). Risk of bias of the included RCTs was not investigated and there are indications that in part simplifying assumptions were used for the meta-analysis that are not justified. For example, multiple outcome measures in the same study population were not corrected for the dependence of effect estimates, which may lead to an overestimation of the precision of the reported effects. Therefore, this result is subject to great uncertainty, i.e. the quality of the evidence is low. Evidence for a small positive effect, SMD Cohen’s d of 0.16 (95% CI: 0.03; 0.29) or an unquantified positive effect for “general cognitive function” (thinking) is available from a total of ten RCTs with 470 patients. This result also shows great uncertainty for the same reasons, so that the quality of the evidence is low.

Conflicting evidence for a positive effect on health-related quality of life of a non-quantified size comes from two RCTs [12], [15], [16], [17] with a total of 214 patients. Only one [12], [17] of the two RCTs showed a statistically significant effect of the intervention. For this study, an assessment of the risk of bias was missing. The other RCT [15], [16] showed a high risk of bias. The quality of the evidence is low. Evidence for an unquantified positive effect on behaviour control came from one RCT [14], [18] with 96 patients. The study has a high risk of bias, so that the quality of the evidence is low. Evidence for a small positive effect, SMD Cohen’s d of 0.19 (95% CI: 0.01; 0.37) on activities of daily living was reported in a meta-analysis [13] of four RCTs with 405 patients. This result also has a low quality of evidence due to the shortcomings of the statistical methods of the meta-analysis and the lack of an assessment of risk of bias in the studies.

The effectiveness of the intervention on individual components of cognition (concept formation, planning, flexibility) cannot be proven with the available evidence. Five RCTs [11], [15], [19], [20], [21], [22], [23] with a total of 202 patients found no effect. The RCTs have a high risk of bias and the quality of the evidence is low. Likewise, no effect was found for the outcome measures of self-efficacy (one RCT [15], [16] with 98 patients, high risk of bias) and social participation (two RCTs [14], [15], [16], [18], 194 patients, high risk of bias). The quality of the evidence for both outcomes is low.

The evidence for the outcome measures of attention (three RCTs [15], [16], [24], [25], 126 patients, high risk of bias) and memory (four RCTs [11], [15], [21], [23], [24], [26], 188 patients, unclear and high risk of bias) is contradictory, but mostly found no effect. The quality of evidence for both outcomes is low.

A stratification of the effectiveness of occupational therapy, separated according to the type of intervention (inpatient and outpatient), was not possible, as the majority of the included systematic reviews do not provide any information on the care setting or summarize the evidence across the board.

In a supplementary presentation, we summarise the evidence comparing occupational therapy with a cognitive component to occupational therapy without a cognitive component to give an overview of studies that examine the contribution of specific cognitive function training to the effect. Assessments of risk of bias were not extracted, therefore we do not assess the quality of the evidence. A total of seven systematic reviews [11], [12], [14], [27], [28], [29], [30] with seven RCTs and 407 subjects were included for this comparison. A positive effect of occupational therapy with a cognitive component compared to occupational therapy without a cognitive component cannot be proven on the basis of the available evidence. Evidence from one RCT is available for a positive effect on concept formation [11], [31], while no effect was found for verbal fluency [30], [32], time judgement [28], [33], and gesture imitation skills [29], [34]. Conflicting evidence from three RCTs [11], [28], [29], [31], [33], [34] is available for the effect on functioning in daily life activities, while evidence from two RCTs [12], [27], [35], [36] with 117 subjects found no effect on the health-related quality of life.

In a further supplementary presentation, this overview also summarises the evidence on the comparative effectiveness of different forms of occupational therapy for cognitive impairment. Five systematic reviews [11], [12], [14], [15], [30] with 20 RCTs and 853 patients were included for this comparison. Based on the available evidence, no statement can be made that certain interventions are more effective than others, because some of the available meta-analyses find small to moderate effects, but these are not statistically significant. Individual RCTs that report positive effects have very small sample sizes, and the reporting regarding statistical uncertainty in the systematic reviews is imprecise, so that no evidence for a positive effect can be derived from them. Furthermore, an assessment of the quality of the evidence also requires a risk of bias assessment, which we did not perform in the context of a supplementary presentation.

#### Quality of the evidence

The methodological quality of the nine included systematic reviews was examined with the AMSTAR 2 instrument [10], which asks for a total of 16 criteria. On average, the systematic reviews had five ratings of “no” (criterion not met) with a range of four to eleven. Common shortcomings (rating “no” in four systematic reviews or more) were that no study protocol had been prepared in advance (four systematic reviews [13], [27], [28], [29]), that the choice of study types included in the selection were not justified (eight systematic reviews [11], [12], [13], [15], [27], [28], [29], [30]), that the literature search was not comprehensive (seven systematic reviews [11], [12], [13], [15], [29], [30]), that the excluded studies were not specified in a list with reasons for exclusion (six systematic reviews [12], [13], [14], [15], [29], [30]), that the funding of the primary studies was not reported (seven systematic reviews [11], [12], [13], [14], [15], [28], [29]) and conflicts of interest for the systematic review itself were not specified (six systematic reviews [12], [13], [14], [15], [27], [30]). In contrast, with regard to the categories research question, study selection, and data extraction the included systematic reviews met the AMSTAR 2 criteria, except for one systematic review in each section. In four of the systematic reviews [12], [14], [15], [30], the results for the outcomes were not described quantitatively with effect measure and uncertainty measure, but only qualitatively (e.g. “there was an improvement”). This severely limits the assessment of clinical relevance and precision (statistical uncertainty). A further challenge to the assessment of the evidence in the present overview was that three of the systematic reviews [12], [13], [30] did not assess risk of bias for the RCTs and that the risk of bias assessment in one of the Cochrane reviews [11] did not use the adequate randomisation item and combined the items on blinding, making this difficult to assess. The systematic review by Park et al. [13] with twelve AMSTAR 2 criteria not met performed the worst. Since the meta-analyses for the cognitive and other outcome measures that found the small positive effect of occupational therapy compared to no occupational therapy are based on this systematic review, this limits the strength of the evidence and increases the uncertainty that the small positive effect could also be based on systematic bias. This is because the systematic review by Park et al. [13] did not examine the risk of bias of the RCTs included in the meta-analysis, therefore the information is missing for nine RCTs. Risk of bias assessment of RCTs by the included five systematic reviews was only available from three systematic reviews [11], [14], [15] for ten out of 20 RCTs. These ten RCTs have predominantly high or unclear to high risk of bias. Six RCTs can be attributed an overall high risk of bias because either the randomisation sequence was inappropriately generated or there was no concealed allocation to the groups, or because both were the case, or because two other risk of bias items had a high risk. Four RCTs had a total of at least one unclear to high risk of bias because either data were missing or one of the other bias items also had a high risk of bias. 

Overall, the sample sizes of the studies were small. The median number of sample size was 40 subjects with a range between 12 and 334. This means that most of the studies have a limited statistical power and only relatively large effects can achieve statistical significance.

It is also problematic that the method for the meta-analysis in Park et al. [13] is not described in detail, especially since the authors included several outcome measures from one study in each meta-analysis and there is no indication of the use of adequate correction procedures for the dependence of the data. Since similar measurement instruments or subscores are often positively correlated, this would lead to an overestimation of precision, and thus the small positive, statistically significant effect is also in question. 

### Discussion

#### Applicability of findings to German-speaking countries

No intervention studies in the healthcare systems of German-speaking countries were found in the identified reviews. However, as the application of occupational therapy in everyday practice is influenced by local treatment patterns, remedy guidelines, and local professional organisations, it may not be justified to transfer the results to the German-speaking setting.

#### Limitations

This work has several limitations. Occupational therapy is a complex intervention consisting of several components. It is characterised by the fact that its aim is not only to improve individual functions, but to improve the performance of everyday activities. The selection of relevant everyday activities is made together with the patient. Often, different treatment methods are combined (e.g. compensatory methods and such interventions that address the recovery of specific functions), so that different interventions can be included under the same definition of “occupational therapy”. Rehabilitation interventions for cognitive disorders caused by the CNS diseases we are looking at use occupational therapy together with other therapeutic interventions, such as drug therapy and physiotherapy. The estimation of the effects of occupational therapy is therefore problematic, since the multidisciplinary integration of occupational therapy can influence the effect. The same applies, to some extent, to possible comparative interventions in rehabilitation with or without cognitive components. This complex definition of occupational therapy and the comparative therapies already poses a challenge in the design, conduct, and interpretation of primary studies, but confronts systematic reviews with additional obstacles because of the further reduction in information provided by systematic reviews compared to primary studies.

Thus, the results on the outcomes are reported across indications. However, a summary of clinical efficacy across causes of illness seems justified in that occupational therapy is often provided in mixed groups with patients after stroke and patients with traumatic brain injury. This indicates that similar limitations are present and similar forms of therapy are applied.

One difficulty with this overview was to include all reviews in which results on RCTs of occupational therapy interventions with a cognitive aspect can be found, but on the other hand to stick to the methodology of the overview, which takes its statements exclusively from the included reviews without resorting to the primary studies. Ultimately, both the completeness of the evidence and the accuracy of the synthesis suffer from this format. 

The expectation in selecting the methods for this overview was that we would find a large number of good quality systematic reviews on our topic. This impression came from the systematic reviews and overviews already listed in the Cochrane Database on the diseases we were looking for (stroke, traumatic brain injury, multiple sclerosis, Parkinson’s disease), on the one hand, and on cognitive rehabilitation and occupational therapy, on the other. In the end, this expectation could not be fulfilled. Some cognitive rehabilitation measures have been included in the therapeutic treatment methods of occupational therapy in recent years. However, occupational therapy should still be seen as a separate form of treatment, even though it adopts cognitive methods from neuropsychology. In some reviews, occupational therapy is defined as a treatment used by occupational therapists, and in others as occupational therapy treatment catalogues. The present overview includes these definitions, as well as descriptions that suggest occupational therapy character, such as “occupation-oriented rehabilitation”. Systematic reviews were also included if they did not focus exclusively on occupational therapy treatments, but were nevertheless included and labelled as such. 

However, many systematic reviews had to be excluded because they did not specify whether the cognitive rehabilitation methods studied should be considered occupational therapy.

Another limitation was that occupational therapy is often a part of multidisciplinary rehabilitation, and often the multidisciplinary intervention is the subject of reviews without the contribution of occupational therapy being identifiable. These reviews or primary studies also had to be excluded. 

Other reviews found in the preliminary literature selection process met the criterion of disease area and treatment with occupational therapy, but ultimately did not contain a description suggestive of individuals with cognitive impairment, and therefore had to be excluded. This overview also included studies that did not explicitly address cognitive impairment in the study population, but did use cognitive interventions or did report cognitive outcome measures. Cognitive impairment may be the reason why activities of daily living, such as dressing or walking, are impaired. Therefore, activities of daily living may be the only endpoint for cognitive interventions. Studies of this type are included in this overview because cognitive interventions suggest that at least some patients had cognitive impairment. Our definition is, therefore, somewhat vague.

The definition of occupational therapy and its differentiation from other, e.g. neuropsychological interventions in cognitive rehabilitation was done based on the evidence of the authors of the systematic reviews. A large number of reviews on cognitive rehabilitation are available – but they do not refer to occupational therapy, or they consider occupational therapy as a method of cognitive rehabilitation. It cannot be ruled out that other rehabilitation interventions were not considered that would certainly have been suitable for inclusion in an occupational therapy program. 

In addition to Cochrane reviews, this overview also includes other systematic reviews that differ from the Cochrane reviews in their methodological approach. In particular, systematic reviews were included that did not provide quantitative information on effects and their statistical uncertainty, as well as systematic reviews that had not conducted a risk of bias assessment of the primary studies. In addition to the disadvantage of not being able to conduct a separate meta-analysis due to the reduced information, this makes it impossible to assess the confidence of the overall evidence comprehensively on an outcome measure, as can be done with the GRADE methodology [37], for example. We used the language of GRADE (high, moderate, low confidence in evidence) for the main comparison of occupational therapy versus no occupational therapy in the overall assessment of the evidence per outcome measure, even though we could not use GRADE due to the lack of information and our assessment shows reduced quality because of lacking information. Thus, considering systematic reviews with different methodologies limits comparability and information on the quality of evidence, but, on the other hand, also limits the loss of available evidence. 

The supplementary presentation of the two comparisons of different forms of occupational therapy was not part of the pre-defined research question. The intention here was to provide a first exploratory overview of the therapy comparisons, which becomes particularly relevant in the situation where occupational therapy is increasingly an already established form of therapy. We did not extract risk of bias assessments, or conduct an assessment of the overall evidence per outcome measure or the quality of the evidence.

The included meta-analyses used standardised effect sizes as effect measures. To assess whether the effect sizes found were clinically significant effects, Cohen’s rule of thumb [38] was used to classify small (0.2), medium (0.5), and large (0.8) effects. This classification would first have to be validated for the range of the present diseases and is only to be understood as a guide because a categorisation of the clinical relevance of effect sizes has to be evaluated by the impact on patients.

In order to limit the scope of this review, interventions and outcome measures for visuospatial impairments and perceptual impairments were not considered, although these also belong to the domain of cognition. We thus followed previous reviews by Hoffmann et al. [39], and Reinsperger et al. [29]. The outcome fatigue was also not included, although this endpoint can also be considered a cognitive endpoint. This is also in line with similar work [40].

The fact that positive effects of the interventions are difficult to prove could be due to the great clinical heterogeneity of the included studies, which are summarized in this overview: the diversity of the interventions, the type and severity of the present brain injuries and limitations, the age of patients as well as the environment of the interventions (hospital, own home) and parallel treatment with medication and other forms of therapy. It can be assumed that the variation in effects is large as a result.

The present overview cannot include this level of detail and cannot conduct an investigation of heterogeneity. However, even at the level of primary studies, the small number of studies limits an investigation of the influence of heterogeneity.

The literature search on which the overview is based limited the publication languages to German and English. This may have led to relevant studies not being identified. However, since at the same time the applicability of the evidence found to the health care systems in German-speaking countries should be given, the restriction of the publication language seems less relevant.

Furthermore, it cannot be ruled out that small studies without statistically significant effects were not published at the level of the primary studies (publication bias), which would lead to an overestimation of a positive effect, especially in meta-analyses.

## Evaluation of costs and cost-effectiveness

### Methods

#### Main inclusion and exclusion criteria for systematic reviews

The same inclusion and exclusion criteria for study population, intervention, and comparator as for the assessment of clinical efficacy were used to assess the costs and cost-effectiveness of occupational therapy for cognitive disorders. Outcome measures include changes in resource usage, additional costs of occupational therapy, and additional costs per life year gained or quality-adjusted life years gained. All health economic study types are included. The evaluations must relate to German-speaking countries.

#### Literature search, selection, assessment of study quality, data extraction, and evidence synthesis

The search included the same databases and the same search period as the search for clinical efficacy. The assessment of methodological quality only takes place with regard to the question of whether a comprehensive, reproducible literature search and selection took place. For this purpose, the AMSTAR 2 instrument is used in a modified form. The extraction of the results for the included systematic reviews is carried out analogously to the assessment of clinical efficacy. However, instead of the outcome measures for clinical efficacy, the health economic outcome measures are now extracted from the reviews.

### Results and discussion

No suitable systematic reviews on the costs or cost-effectiveness of occupational therapy in adult patients with cognitive deficits due to neurological diseases (except dementia) were identified. Therefore, no conclusions can be made about costs and cost-effectiveness.

## Evaluation of patients and social aspects

### Methods

#### Main inclusion and exclusion criteria

The inclusion criteria for systematic reviews on patient and social aspects correspond to the inclusion criteria for clinical efficacy for the target population and intervention. However, studies with any comparative intervention and non-comparative studies could have been included. The outcome measures are based on the questions (“assessment elements”) of the HTA Core Model^®^ of the European network for Health Technology Assessment [41]. They are: utilisation, knowledge, attitude, acceptance, satisfaction, experiences, expectations of the treated, as well as access to occupational therapy, type and extent of communication, and information on occupational therapy and its evaluation by patients. The outcome measures could be reported from the perspective of patients, from the perspective of their caregivers or family members, or from the perspective of the treating professionals. 

#### Literature search, selection, assessment of study quality, data extraction, and evidence synthesis

The literature search already described above was supplemented by a literature search in the databases PsycINFO as well as CINAHL for the period from January 1, 2010, to June 17, 2020. All systematic reviews were screened by one person with regard to the inclusion criteria and reviewed by a second person. Data on study characteristics and patient and social aspects of occupational therapy were tabulated by one person and the results were reviewed by a second person. Differences were resolved by discussion. To assess methodological quality, the AMSTAR 2 instrument was used in a modified form. Only criteria that verify whether a comprehensive, reproducible literature search and selection has taken place were applied.

### Results

No systematic reviews were identified that met all inclusion criteria. Five systematic reviews [42], [43], [44], [45], [46] were identified on patient and social aspects, such as experiences with illness, therapy, and the treating professionals, in people with stroke or traumatic brain injury or their family caregivers. The systematic reviews either did not report the type of therapy or generally characterised it as rehabilitation. Cognitive deficits were not an inclusion criterion in the identified systematic reviews and were not explicitly investigated. We assume that the experiences of patients with stroke or traumatic brain injury may be partly influenced by cognitive deficits, even if these are not explicitly mentioned and, therefore, may also be applicable to patients with cognitive deficits after damage to the central nervous system. Hence, these five systematic reviews were described in a supplementary presentation. Three systematic reviews [44], [45], [46] examined aspects from the perspective of patients who had a stroke, one [42] from the perspective of working age people with an acquired brain injury who wish to return to work, and one [43] from the perspective of caregivers of patients who had a stroke. Outcome measures were experiences with rehabilitation (two systematic reviews [43], [45]), once from the patients’ perspective and once from the family carergivers’ perspective. One systematic review looked at patients’ views on the impact of a stroke on roles and the “self” [46], another at experiences of occupational identity disruption post stroke [44], and a third at factors experienced as important for returning to work [42]. Three systematic reviews applied thematic syntheses [43], [45], [46]; two systematic reviews applied ethnographic syntheses [42], [44] of qualitative studies. The number of included studies ranged from 10 to 33 with numbers of subjects between 111 and 465. The qualitative studies in the systematic reviews predominantly used qualitative interviews as a data collection instrument. Methodological quality was assessed using the modified AMSTAR 2 checklist with eleven criteria. The median of “no” ratings (criterion not met) in the systematic reviews was five with a range of two to six. Common deficiencies (rating “no” in three systematic reviews or more) were: no study protocol had been prepared in advance (three systematic reviews), the reasons for exclusion were not stated for the excluded studies (five systematic reviews) and the funding of the primary studies was not reported (five systematic reviews). In contrast, the systematic reviews largely fulfilled the criteria in the categories research question, selection of study type, assessment of study quality and indication of conflicts of interest. 

The following aspects were highlighted by the analyses and data syntheses:

Peoples et al. [45] examined how people experienced inpatient rehabilitation after a stroke. They identified an overarching theme of power and empowerment with six subcategories: (1) coping with the new situation, (2) need for information, (3) physical and non-physical needs, (4) being personally valued and treated with respect, (5) working with professionals, and (6) taking responsibility and control. 

Satink et al. [46] examined the question how patients who had suffered a stroke experience the impact of the disease on their roles and selves. Thematic analysis revealed seven descriptive themes: (a) ‘I am only half a person’, (b) struggle with role discontinuity, (c) uncertainty after discharge, (d) desire to regain role continuity, (e) hope to move on and adapt, (f) moving from passive to active in context, and (g) the gap between patients who had had a stroke and health professionals. From this, three overarching analytical themes were developed: (1) managing discontinuity is a struggle, (2) regaining roles: to continue or to adapt?, and (3) context influences management of roles and self.

Martin-Saez et al. [44] used the systematic review to develop a model for the disruption of occupational identity in stroke patients. The model includes three phases: what factors cause occupational identity disruption, the experience of occupational identity disruption, and coping with occupational identity disruption. The four factors responsible for identity loss in phase one are (1) an externalised and fragmented body, (2) loss of control, freedom, and independence, (3) loss of participation in occupations, and (4) a change in social and family interactions. Phase two is characterised by a loss of self-esteem and doubts about one’s identity. In phase three, there is usually a reduction in social relationships followed by reconstruction, partly through alternative ways of enjoying previous occupations.

Frostad et al. [42] investigated which factors are perceived by persons with acquired brain injury to be helpful in returning to work. They identified four key concepts for returning to work from the 16 studies: (1) empowerment, (2) self-awareness, (3) motivation, and (4) support.

Luker et al. [43] examined experiences, needs, and preferences of family caregivers during inpatient rehabilitation of patients who had a stroke. They identified seven analytic themes: (1) overwhelmed by feelings, (2) recognition as a stakeholder in the recovery process, (3) desire to be heard and informed, (4) insisting on action and outcomes, (5) being legitimate clients, (6) navigating a foreign culture and environment, and (7) managing the transition home.

### Discussion

Essentially, after a stroke or a traumatic brain injury both the patients and the caregiving relatives seem to go through certain phases during rehabilitation and after discharge, during which health professionals can contribute more or less to successful coping. First, patients experience a loss of their abilities and a discontinuity with their former life at the level of body, ego, and roles. This is followed by reassessment, readjustment, a regaining of roles, and, finally, a continuation of one’s own life. A decisive turning point is the discharge home. It is associated with a transition to increased control and self-management by the patient, but also with the loss of the protected, supportive environment in the rehabilitation facility and with uncertainty. Regaining an active, self-determining role is a process that requires therapists to find the right level of support from patients and relatives.

Patients in the studies wanted individual and sufficient information, especially about the cause of the disease, the individual progress, the evaluation of the treatment plan and decisions about discharge and aftercare. They wanted more attention to be paid to their psychosocial needs: regaining their roles, the practical needs of managing at home, preparing for a return to work, adapting to life situations, and transitioning to independence.

In the studies, relatives wanted to be understood and involved as stakeholders in the patient’s recovery process and also wanted to receive the information they needed from professionals in order to do so, such as: information on prognosis of recovery, assessment outcomes, rehabilitation and therapy, how best to support and manage the stroke survivor, sexuality after stroke, and preparation for discharge. The information was to be offered in different formats, individually tailored and jargon-free. They wanted to be contacted proactively.

In principle, it is in line with the client-centred approach of occupational therapy to take wishes into account. 

#### Limitations

The present systematic review has several limitations. We could not identify any systematic reviews that examined patient and social aspects specifically regarding occupational therapy for adult patients with cognitive deficits due to neurological disorders of the central nervous system. The systematic reviews that we consider partially transferable to our target population, and which we have therefore included in a supplementary presentation, do not differentiate between the forms of therapy in rehabilitation and do not limit their study populations to people with cognitive deficits. However, it must be assumed that not all systematic reviews to which the extended inclusion criteria apply were found. The search strategy used was linked to terms for occupational therapy, and systematic reviews that only specified rehabilitation as an intervention were, thus, not found. A new search and reselection of significantly more hits for the expanded topic was not feasible considering the given resources and would also have gone beyond the research question posed.

The quality of the included systematic reviews was good in that they had clear questions, comprehensible inclusion criteria, well-described included studies, as well as comprehensible results. Above all, shortcomings existed in the duplicate study selection and data extraction.

However, both the systematic reviews and the patients of the included studies did not come from Germany or German-speaking countries, but from Northern Europe and the Anglo-Saxon countries. Therefore, it is questionable to what extent the experiences of patients and relatives in particular can be transferred to the specialist staff and the rehabilitation facilities. 

Qualitative and quantitative studies, e.g. surveys and interviews, should be conducted in German-speaking countries to find out whether the needs and preferences of patients and their relatives are similar to those in the studies from Northern European and Anglo-Saxon countries. At the same time, possible barriers to client-centred care should be investigated at the micro, meso and health system levels so that appropriate interventions can be developed to improve care.

## Evaluation of ethical aspects

### Methods

#### Main inclusion and exclusion criteria

The inclusion criteria for systematic reviews assessing ethical aspects correspond to the inclusion criteria for clinical efficacy for the target population and intervention, but different criteria were used for the comparator. Studies with any comparative intervention and non-comparative studies could be included. Outcome measures are ethical aspects such as morality, conscience, responsibility, justice, autonomy, equity, fairness, informed consent, and human rights. In addition to systematic reviews, professional ethical standards and codes or other documents containing frameworks with criteria for addressing ethical issues for occupational therapists are included.

#### Literature search, selection, assessment of study quality, data extraction, and evidence synthesis

The literature search was based on the same databases used to search for clinical efficacy and patient and social issues. In addition, an internet search was conducted. The sources found were screened by one person and checked for their relevance to ethical questions. The selection was reviewed by a second person. Identified frameworks with criteria for weighing ethical aspects in occupational therapy and the ethical principles underlying them are described. Specific ethical aspects of occupational therapy for people with cognitive impairment due to neurological conditions were tabulated from the included documents by one person and categorised according to the identified normative frameworks. A second person checked the evaluation. Discrepancies were resolved by discussion.

### Results

#### Ethical principles in occupational therapy

The search for professional codes of ethics and guidelines via the websites of the professional associations and professional societies of occupational therapists in German-speaking countries and of European and worldwide umbrella organisations, resulted in six documents [47], [48], [49], [50], [51], [52] from which the ethical principles and problem solving models for dealing with ethical conflicts that the professional associations of occupational therapists consider relevant for their professional practice can be taken.

The professional codes refer to the four principles of biomedical ethics according to Beauchamp and Childress [53]: respect for autonomy, non-maleficence, beneficence, and justice. However, they add other principles, sometimes with variations between different organisations: utility and integrity, as well as collegiality and professionalism. The principle of utility addresses the level of the population and can be interpreted as maximising the benefit for the population (“to provide the best available and acceptable solution for the largest population”) but has not been adopted by the German Occupational Therapy Association. Integrity is understood as the integrity of the professional. It includes honesty, confidentiality, and, in the problem solving model of the German Occupational Therapy Association [48], reliability. 

A problem solving model for ethical conflicts was developed by the European umbrella organisation COTEC [50], adopted by Ergotherapie Austria and adapted as well by the German Occupational Therapy Association on the basis of another model. In both models, after identifying the problem possible courses of action are identified, the advantages and disadvantages of their consequences are named and then each is assigned to the relevant ethical principles that could possibly conflict with one another. This should make it easier for occupational therapists to make conscious value decisions.

#### Ethical aspects of occupational therapy treatment

Three systematic reviews [54], [55], [56] were identified on specific ethical aspects for adult patients with cognitive impairment. From these, 16 ethical aspects were identified that were relevant for occupational therapists.

The autonomy of the patients and their restriction due to the consequences of the disease, as well as the resulting tensions with and for the therapists, are the central theme of the ethical analysis. Due to sensomotoric, communicative and/or cognitive limitations, the patients’ ability to express themselves may be restricted. Cognitive limitations and a restricted perception of their own deficits can, furthermore, especially affect their ability to make decisions. This is a particular challenge for occupational therapy professionals because client-centred goal setting is a central professional standard of the professional self-image of occupational therapists. The opposite situation can also occur when the continuing (limited) decision-making competence is not included in health-related decisions. An attempt is made to take into account the loss or regaining of autonomy on behalf of the patient, firstly by considering rehabilitation as a process of regaining autonomy and consciously handing over decision-making competences to the patient, but also secondly by trying to involve patients in the phase of limited decision-making competence, or at least to find out their preferences by other existing means, e.g. by involving relatives. It is also pointed out that, in the dependency phase, the patient’s perception of autonomy depends on the relationship between the patient, the caregiver and the environment. Furthermore, it is pointed out that the integration of disabilities and limitations into the patient’s identity is an important goal of rehabilitation and serves the patient’s self-realisation.

In the ethical aspects listed, the autonomy principle can come into conflict with the principle of beneficence, the principle of non-maleficence and the integrity, as well as the professional self-image, of occupational therapy professionals.

### Discussion

The autonomy of the patient and its restriction due to the consequences of the disease, as well as the resulting tensions with and for the therapists, are the central themes of the ethical analysis. Due to sensorimotor, communicative and/or cognitive limitations, the patients’ ability to express themselves may be restricted. Cognitive limitations and a restricted perception of one’s own deficits can also especially affect the ability to make decisions. This is a particular challenge for occupational therapy professionals because client-centred goal setting is a central professional standard of the professional self-understanding of occupational therapists. In the present article, we were only able to identify an ethical analysis of one specific cognitive deficit: “unawareness” about one’s own deficits in patients with acquired brain damage. Here, studies comparing the different intervention strategies would be desirable: whether a reduction of “unawareness” should be aimed at first before therapy goal-setting is carried out with the patient and when the appropriate time for this would be; or whether client-centred goal-setting should be carried out immediately, but possibly splitting it into realistic short-term and more unrealistic long-term goals, or whether it should be carried out with the help of relatives.

In addition, a systematic review of primary studies on ethical aspects in occupational therapy for patients with cognitive deficits should be conducted. If appropriate, studies could also be included here that deal generally with the rehabilitation of cognitive deficits in the included indications because it can be assumed that many aspects are transferable to occupational therapy. 

#### Limitations

The present overview on ethical aspects of occupational therapy in patients with cognitive deficits as a result of neurological diseases has some limitations. We did not find any systematic reviews on ethical aspects in this target population in the bibliographic databases. Concerning the internet search, very few empirical studies with patients with cognitive deficits and occupational therapy treatment were available in the systematic reviews, too. This might have been partly due to the limited search on the internet. Thus, we have relatively detailed recommendations on ethical problem solving in occupational therapy, but few empirical examples. In addition, the systematic reviews had only rudimentary descriptions, if any, of the included studies. However, it could be seen that all primary studies had been conducted outside the German-speaking area and, therefore, the transferability to the healthcare system in German-speaking countries must be questioned. 

## Conclusions and recommendations

### Recommendations for practice and research

Based on this systematic overview, it cannot be demonstrated with certainty that occupational therapy for cognitive impairment is an effective therapy for adult patients with central nervous system injuries. The available evidence shows a small positive effect on cognitive functioning, functioning in activities of daily living and health-related quality of life compared to not using occupational therapy. However, the confidence level of the evidence is low as far as can be understood from the overview methodology. In an exploratory supplementary presentation, which included comparisons of different forms of occupational therapy with and without cognitive components, there were predominantly no statistically significant differences between the forms of therapy for cognitive performance enhancement. An assessment of the risk of bias was not carried out in the supplementary presentation.

On the other hand, the efficacy of occupational therapy cannot be ruled out either because most of the included studies have a relatively small sample size and limited statistical power, and, therefore, smaller effects may not be demonstrated. In addition, the heterogeneity of study populations, interventions, and comparisons suggests a high variance of effects. Since none of the included studies were conducted in the health care systems of the German-speaking countries, the transferability of the results also appears questionable. 

Based on these results, we recommend the continuation of occupational therapy research RCTs with patient-relevant outcomes and with a sufficient sample size. For future RCTs on occupational therapy, the exact definition and description of the form of occupational therapy investigated is important, as well as the comparability of the concomitant therapies carried out in parallel in the intervention and control groups. We also recommend the implementation and registration of well-planned observational studies in the care routine. In the study design of such studies, an appropriate collection of potential confounders should be taken into account, for which adjustments can be made in the analysis.

Future studies should also integrate health economic components on resource use and costs.

Systematic reviews can especially support the planning of new studies. For future systematic reviews, instead of conducting an overview, we recommend going back to the primary studies and adding a new search for primary studies in each disease area to the primary studies already assessed in Cochrane Reviews and HTA reports of similar methodology.

With regard to patient and social aspects, both patients after a stroke or a traumatic brain injury and their family caregivers seem to go through certain phases during rehabilitation and after discharge. In this process, medical professionals can contribute to a greater or lesser extent to successful coping. In qualitative studies from Northern European and Anglo-Saxon countries, patients and relatives have expressed wishes for better communication and more client-centred rehabilitation measures, which may generally be assumed to be shared by patients and relatives in German-speaking countries as well. Moreover, the expressed wishes are in line with the understanding of client-centred treatment in occupational therapy. Therefore, in addition to verifying the needs of patients and relatives during and after rehabilitation through surveys of patients and relatives in German-speaking countries, investigations should also be carried out into what hinders the implementation of client-centred therapy in patients with cognitive deficits after neurological diseases of the central nervous system and what interventions could promote it.

In the present article, some solution models for ethical conflicts as well as16 ethical aspects could be identified, but there were only few systematic reviews on ethical aspects from the perspective of patients, relatives, and professionals based on empirical primary studies. Therefore, a systematic review of primary studies on ethical aspects in occupational therapy for people with cognitive deficits, their relatives, and professionals should be conducted. If appropriate, studies could also be included here that deal generally with the rehabilitation of cognitive deficits in the included indications because it can be assumed that many ethical aspects are transferable to occupational therapy. 

## Notes

### HTA report

This article is the short version of the HTA report of the same title [9].

### Competing interests

The authors declare that they have no competing interests.
